# Programming Plyometric-Jump Training in Soccer: A Review

**DOI:** 10.3390/sports10060094

**Published:** 2022-06-10

**Authors:** Rodrigo Ramirez-Campillo, Jason Moran, Jon L. Oliver, Jason S. Pedley, Rhodri S. Lloyd, Urs Granacher

**Affiliations:** 1 Exercise and Rehabilitation Sciences Laboratory, School of Physical Therapy, Faculty of Rehabilitation Sciences, University Andres Bello, Santiago 7591538, Chile; rodrigo.ramirez@unab.cl; 2 School of Sport, Rehabilitation and Exercise Sciences, University of Essex, Essex CO4 3SQ, UK; jmorana@essex.ac.uk; 3 Youth Physical Development Centre, Cardiff School of Sport and Health Sciences, Cardiff Metropolitan University, Cardiff CF23 6XD, UK; joliver@cardiffmet.ac.uk (J.L.O.); jpedley@cardiffmet.ac.uk (J.S.P.); rlloyd@cardiffmet.ac.uk (R.S.L.); 4 Division of Training and Movement Sciences, University of Potsdam, Am Neuen Palais 10, Building 12, 14469 Potsdam, Germany

**Keywords:** human physical conditioning, exercise, resistance training, muscle strength, plyometric exercise, musculoskeletal and neural physiological phenomena, movement, sports, football, youth sport

## Abstract

The aim of this review was to describe and summarize the scientific literature on programming parameters related to jump or plyometric training in male and female soccer players of different ages and fitness levels. A literature search was conducted in the electronic databases PubMed, Web of Science and Scopus using keywords related to the main topic of this study (e.g., “ballistic” and “plyometric”). According to the PICOS framework, the population for the review was restricted to soccer players, involved in jump or plyometric training. Among 7556 identified studies, 90 were eligible for inclusion. Only 12 studies were found for females. Most studies (n = 52) were conducted with youth male players. Moreover, only 35 studies determined the effectiveness of a given jump training programming factor. Based on the limited available research, it seems that a dose of 7 weeks (1–2 sessions per week), with ~80 jumps (specific of combined types) per session, using near-maximal or maximal intensity, with adequate recovery between repetitions (<15 s), sets (≥30 s) and sessions (≥24–48 h), using progressive overload and taper strategies, using appropriate surfaces (e.g., grass), and applied in a well-rested state, when combined with other training methods, would increase the outcome of effective and safe plyometric-jump training interventions aimed at improving soccer players physical fitness. In conclusion, jump training is an effective and easy-to-administer training approach for youth, adult, male and female soccer players. However, optimal programming for plyometric-jump training in soccer is yet to be determined in future research.

## 1. Introduction

Soccer is a very popular sport, with an estimated 270 million participants around the world [1], including an estimated 60 million female players by 2026 [2], and 5 billion fans [3]. Success in soccer requires a myriad of factors [4] such as well-developed physiological and physical fitness applicable to female [5], male [6], and youth athletes [7,8,9]. A range of training methods designed to improve soccer players’ physical fitness have been reported in the scientific literature. One of the most popular methods is jump or plyometric training [10], usually involving eccentric–concentric muscle actions, with a rapid transition (e.g., amortization) between these actions, commonly termed the stretch-shortening cycle. In the context of this paper, plyometric training is defined as the systematic use of any form of jumping as a primary mode of training. Jump training seems to be equally, or even more, effective compared to other training methods (e.g., traditional resistance training) for the improvement of several physical fitness outcomes such as sprinting, jumping, and change of direction [11,12]. Furthermore, jump training requires little or no equipment to perform and therefore presents a cost-effective training method for the improvement of a range of different athletic qualities [13]. Jumping exercises also mimic the specific short-duration high-intensity actions of soccer, potentially increasing the transference effect between jump training exercises and on-pitch soccer performance [14,15,16].

Despite the publication of a considerable volume of jump training literature involving soccer players, very few studies have attempted to elucidate the optimal training programming parameters such as duration [17], frequency [18], type of jump exercise [14,19], volume [20], intensity [21], recovery (inter-repetition recovery; inter-set and inter-exercise recovery; inter-session recovery) [22,23,24], progressive overload [25], taper strategies [26], type of surface [27], or the effects of jump training combined with other training methods [28]. Moreover, most studies investigating jump training in soccer involved only small samples of participants (i.e., n = 10) [11,12,29] which is a common problem in sport science literature using highly trained athletes [30]. In an attempt to address sample size issues within the existing literature, systematic reviews with and without meta-analyses have been conducted in adult male [31] and female players [32], as well as in young players [33,34]. However, systematic reviews with and without meta-analyses usually include only randomized controlled trials. Such a research design can be logistically difficult to conduct in highly trained athletes and could, therefore, exclude much of the evidence available in soccer athletes. In addition, a more comprehensive analysis regarding the potential effects of jump training on soccer players’ adaptations may be limited by strict inclusion criteria inherent to systematic reviews with and without meta-analyses [35,36,37]. In this scenario, the analytical latitude offered by a qualitative review might offer an advancement in the field.

Two previous reviews [11,12] have addressed jump training programming issues. However, these studies included athletes with different sporting backgrounds (e.g., soccer, basketball, and volleyball), potentially making the results inapplicable for a particular sport, such as soccer. Indeed, the adaptive responses to jump training may be affected by moderators such as training background [38,39]. Additionally, the number of jump training-related publications has increased 25 fold between 2000 and 2017 [11], with soccer-related studies at a rate of approximately 100 per year [40]. Such an increasing rate of novel publications calls for constant updates of the literature in the form of reviews. Indeed, in related fields of sport science with high scientific productivity, yearly updates may be necessary [41,42,43,44,45,46,47,48,49]. In recent years, “living literature” review strategies have been recommended to cope with fast-growing fields of knowledge [50]. The main advantage of this approach is that it assumes that new knowledge will appear and allow timely improvements in sport and clinical decision making [50]. To our knowledge, such a potentially relevant method has not yet been applied in the field of jump training and soccer.

Therefore, the aim of this review article was to describe and summarize the published scientific literature as it relates to methodological issues surrounding jump training in soccer players with different fitness levels. More specifically, we extracted data on sample characteristics (e.g., age, sex, and expertise level) and exercise programming variables (e.g., exercise intensity, frequency, and volume) from jump training studies in soccer. Findings from this review will facilitate recommendations for future directions of jump training research in soccer. Practitioners will be provided with relevant information on the implementation of jump training in male and female soccer players of different ages and expertise levels.

## 2. Materials and Methods

A search was conducted in the electronic databases PubMed (including Medline), Web of Science and Scopus, with no date restrictions. After an initial search (April 2017), accounts were created in the respective databases for the lead author. The search was refined in May 2019 and updates were received daily (if available) until the preparation of the manuscript (April 2022). Of note, the final search using the respective search syntax is currently ongoing, as our living search strategy in the electronic databases will allow to provide updates periodically. As previously recommended [51], such updates are planned every five years.

Keywords were collected through experts’ opinion (contacted through Expertscape, considering the world’s top-rated expertise in soccer and plyometric exercise, e.g., https://www.expertscape.com/ex/plyometric+exercise; accessed on 1 April 2018), a literature review, and controlled vocabulary (e.g., Medical Subject Headings: MeSH). The following key words were searched in different combinations using the Boolean operators “AND” or “OR”: “ballistic”, “complex”, “explosive”, “force”, “velocity”, “plyometric”, “exercise”, “stretch”, “shortening”, “cycle”, “jump”, and “training”. Only original peer-reviewed full-text articles written in English were considered. One author (RRC) conducted the initial search. When selecting studies for inclusion, retrieved articles were first screened for duplicates through specialized software (EndNote X8 for Windows, Clarivate Analytics, London, UK). In selecting studies for inclusion, a review of all relevant titles was conducted before examination of the abstracts and full-text versions.

Table 1 shows the complete search strategy (search syntax) for each database and information on the search history.

### 2.1. Inclusion Criteria

According to the PICOS framework, the population for the current review was restricted to soccer (Association football) players (including beach soccer, indoor soccer, futsal, and related modalities), although restrictions on age, sex or initial physical fitness levels were not imposed during the initial search. Included studies were those interventions, of three or more weeks duration, which included jump training exercises as a primary component, individually or embedded into a wider training program. Additionally, in line with previous suggestions [12,52,53], to avoid exclusion of potentially relevant studies, both non-randomized and non-controlled intervention studies were considered as well.

### 2.2. Exclusion Criteria

Although cross-sectional, acute studies related to jump training exercises may offer valuable information for soccer-related athletes and practitioners (e.g., post-activation potentiation; post-activation performance enhancement; delayed-onset of muscle soreness), these were not included in this review. A previous review already addressed a similar topic [54]. However, this review examined the effects of acute exercise interventions in soccer while we scrutinized long-term (≥3 weeks) effects of jump training in soccer. Additionally excluded were review papers or training-related studies that did not focus on the effect of jump training.

### 2.3. Data Extraction

One investigator (RRC) processed all of the collected data. Based on previous recommendations [11,12] and the expert opinion of seven top-10 world-level researchers in the field of jump training scientific literature [55], several data items were considered for extraction in line with previous summaries of jump training research [11,12]. Briefly, the main characteristics of the participants (e.g., age, sex, and fitness level) and jump training interventions (e.g., duration, frequency, and intensity) were extracted.

## 3. Results and Discussion

### 3.1. Study Selection

**Figure 1:**
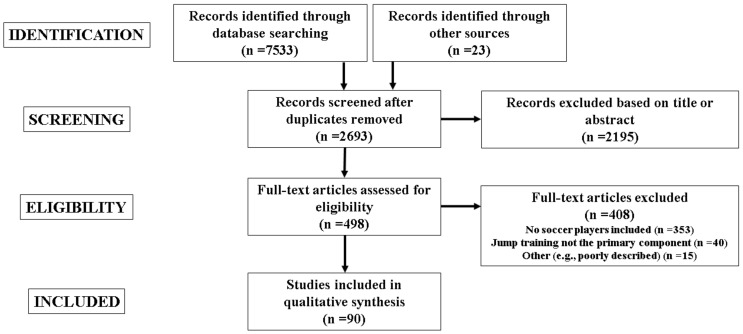
Studies eligibility process.

Among the 7556 identified studies, 4863 duplicates were removed. Following a preliminary screening of titles and abstracts, a further 2195 articles were excluded leaving 498 studies for full-text screening. A further 408 articles were excluded because they did not meet the inclusion criteria. Of note, among the excluded full-text articles, 35 studies incorporated plyometric-jump training. However, these studies included soccer players with players from other sports (i.e., the studies did not compare soccer against other types of sport), thus negating their relevance to the current review. Accordingly, 90 studies were eligible for inclusion with 88 of these conducted in soccer players and two in futsal players. A flow diagram illustrating the eligibility process is presented in Figure 1.

### 3.2. Characteristics of Soccer Players in Jump Training Studies

Among the included studies, 77 reported data for male soccer players and one in male futsal players [56]. Additionally, 11 studies reported data for female soccer players and one with female futsal players [57]. A summary of the characteristics of soccer players in jump training studies is provided in Table 2.

Overall, some trends were noted such as (i) the lack of female data, particularly in youth players; (ii) no study was reported in masters-aged players; (iii) a lack of control groups in ~40% of studies. This is a particular issue in studies in youth in which the effects of biological maturation must be accounted for. A total of 35 studies (from a total of 52) provided some information regarding youth male soccer players’ biological maturity status (e.g., pubertal stage; predicted age of peak height velocity). However, none of the three studies with youth females provided information regarding their biological maturity status. (iv) Furthermore, the majority of studies took place during the in-season period meaning their results may only be applicable to that particular phase of the annual training cycle. This can be useful from an application standpoint but it is also problematic in that participants may also be partaking in other training methods which could provide a similar stimulus to jump training thus making the standardization of training loads difficult. (v) Most studies (i.e., 80%, Table 2) were conducted in individuals who play and/or compete soccer on a recreational level. Most (i.e., 52%, Table 2) of the included studies recruited soccer players without systematic plyometric-jump training experience before the start of the intervention or the authors of the respective studies did not report such relevant information.

### 3.3. Characteristics of Jump Training Interventions

#### 3.3.1. Duration

Among the included studies in this review, the duration of jump training programs ranged between three and 96 weeks with a median of seven weeks with one study reporting an individualized duration. These results are similar to those reported in a previous review of 420 jump-training studies performed in multi-sport cohorts with training duration spanning two to 96 weeks with a mean of 8.6 weeks [12]. Although changes in some study outcomes (e.g., landing impact force) may be achieved even after two weeks of jump-landing training [58] or after three weeks in soccer players (e.g., linear sprint) [15], not all short-duration (i.e., ≤3 weeks) jump training studies in soccer players demonstrated favorable effects on physical fitness variables [59]. Indeed, a meta-analysis regarding the effects of jump training on female soccer players demonstrated greater improvements in jumping height after ≥8 weeks (effect size [ES] = 1.24) compared to <8 weeks (ES = 0.66) [32]. Similarly, among male youth soccer players, better 10-m linear sprint performance improvement was noted after programs >7 weeks (ES = 0.93) compared to ≤7 weeks (ES = 0.11). Moreover, among the few studies in soccer players that incorporated mid-study measurements, although improvements in physical fitness (i.e., linear sprinting; change-of-direction speed; jumping; maximal strength; anaerobic power; ball kicking distance) were noted as soon as four weeks into jump training, larger improvements were observed after periods of 6, 8, 12 and 16 weeks of training [60,61]. Taken together, although the evidence suggests that jump training may induce early adaptations (e.g., after four weeks) in some outcomes of physical fitness in soccer players, including linear sprinting, change-of-direction speed, jumping, maximal strength, anaerobic power, and ball kicking distance, greater improvements may be expected after longer-term interventions. Of note, although the trends in intervention duration are relatively consistent between adult and youth soccer players, no study has compared the relative time-courses of adaptations in youth and adult soccer players’ during a jump training program.

#### 3.3.2. Frequency

Among the included studies, the mean training frequency was two sessions per week, although this also ranged from one to six sessions per week. Few studies in soccer players have investigated the effects of different jump-training frequencies on athletes’ physical fitness. In a recent study [62], prepubertal male soccer players completed either one or two jump training sessions per week for 8 weeks with the total volume equated at 680 jumps. The groups experienced similar improvements in physical fitness (e.g., linear sprint; jumping) under both training frequencies. Similar results were found in amateur female soccer players who completed either one or two jump training sessions per week for 8 weeks (i.e., total volume equated at 810-foot contacts per leg) [18] and in futsal players after 6 weeks of jump training (i.e., total volume equated at 774 jumps) [56]. In line with the aforementioned studies, two meta-analyses [32,33] revealed no effect of jump training frequency on female and young male soccer players’ physical fitness (e.g., linear sprint; vertical jump).

Overall, it seems that when weekly volume is equated, and the total volume is rather moderate (i.e., ~112 jumps per week), training frequency (e.g., one versus two sessions per week) seems not to affect physical fitness adaptations in soccer players, at least in the short term, i.e., ≤8 weeks. However, when a greater volume of jumps needs to be accumulated, a greater training frequency may allow some logistical advantages (e.g., greater inter-repetition rest, and training intensity) that could augment the training response. However, more experimental research is needed to verify the effects of different jump training frequencies as regulators of the effects of other potentially more relevant programming variables such as volume and intensity, particularly among highly trained and lower-level players, from different categories and youth-maturity levels.

#### 3.3.3. Type of Jump Exercise

Most studies that examined the effects of jump exercise type on soccer players’ physical fitness adaptations (e.g., linear sprint; jumping) have been undertaken in male youth players [19,28,63,64,65,66,67,68] with two studies having been carried out in adult male players [15,69]. In general terms, the direction of force application (e.g., vertical vs. horizontal) may affect the degree of adaptation (e.g., physical fitness) of soccer players to jump training. For example, vertical-predominant jump training may have a greater impact on physical fitness outcomes with a greater vertical component (e.g., vertical jump), while horizontal-predominant jump training may have a greater impact on physical fitness outcomes with a greater horizontal component (e.g., linear sprint) [64,70]. This was corroborated in a recent meta-analysis [70], indicating that horizontal jump training was superior to vertical jump training at enhancing horizontal performance. Nonetheless, the same meta-analysis observed that horizontal jump training was at least as effective as vertical jump training at enhancing vertical performance, suggesting that horizontal jump training might be a more efficient method for enhancing multi-vector performance for sport.

Additionally, the type of muscle action (e.g., full stretch-shortening cycle vs. concentric-only movement; fast vs. slow stretch-shortening cycle), may also affect the adaptations of soccer players to jump training, with, for example, fast stretch-shortening cycle jump drills exerting a greater effect on the latter stages of a linear sprint (i.e., maximal speed phase) and slow stretch-shortening cycle jump drills on the earlier stages (i.e., acceleration). Additionally, the load used (e.g., loaded vs. unloaded; force–velocity profile), type of jump-landing support (e.g., unilateral vs. bilateral), the specificity of the jump exercise in relation with the outcome being targeted, and the inter-repetition pattern (e.g., cyclic vs. acyclic), may additionally affect the adaptations (e.g., physical fitness) of soccer players to jump training.

Although there is a reasonable amount of scientific literature on the effects of the type of jump exercise on soccer players’ adaptations, considering the myriad of jump exercise variations that are possible [13,71,72], it is likely that a majority of the jump types that could be incorporated into a soccer training program have not been properly investigated in either isolated or combination formats. This is a research area that warrants further exploration, particularly among female players and adult male players, were less research is available.

#### 3.3.4. Total Program Number of Jumps

The applied total training volume (i.e., number of jumps) across jump training programs varied widely among studies, in part due to the different duration of studies (i.e., 3 weeks vs. 2 years) or the type of jump used (e.g., loaded squat jump vs. jump rope), ranging from 90 up to ~10,000 total jumps, with a median of ~80 jumps per session (range 10–500). However, the optimal values are still yet to be determined, with some interventions prescribing training volume in different ways such as duration, distance, repetitions (i.e., foot contacts; foot contacts per leg) or a mixture of these volume-indexes. To date, very few studies have included jump training groups with different volumes being prescribed to each group. Those that were carried out in male youths [20,67,73,74], and adults [69,75].

Of note, from the aforementioned studies, only three studies in youth [20,73,74] and one in adult male soccer players [75] provided an adequate comparison between groups using different jump volumes. Moreover, only one study was a randomized controlled trial [20]. From these four studies, only one [20] observed greater physical fitness improvements after a greater volume of jump training. The reasons for the different findings are not clear at present though it is interesting to note the results of a recent meta-analysis which demonstrated that stiffness adaptations to jump training were greater when the applied dose was lower [76]. In fact, the duration of the studies we observed (i.e., 6–8 weeks) and the relative difference between the low-high volumes groups included in these studies (i.e., 150–200%) were similar whilst three out of four studies applied the principle of progressive overload. In this sense, further research should be conducted, using adequate study designs, to elucidate the role of jump training volume on soccer players’ physical fitness.

Overall, from the best available evidence (e.g., randomized controlled studies), a low (i.e., ~132 jumps per week) to moderate (i.e., ~236 jumps per week) volume of jumps seems equally effective in improving soccer player’s physical fitness in the short-term (i.e., ~7 weeks). Independent of this, some type of volume-based overload (e.g., number of training sessions per week; training exercises; training sets; training repetitions per set) may be needed to maximize improvements, with a relatively lower volume of jumps at the beginning of the program and a progressive increase in number toward the end. Of note, progressive volume-based overload would need to consider jump intensity as well. For example, a high-volume to low-volume approach might be used when the intensity of jump training is increasing. In some instances, a tapering period may further maximize improvements [26]. From an injury prevention perspective, current evidence points toward the use of a conservative volume, which not only may allow significant physical improvements, but also a lower risk of injury [77,78,79]. Particularly relevant for studies in youth, maturity status might be investigated in relation to the effects of jump training volume from an injury prevention perspective and a physical fitness and motor competency perspectives.

#### 3.3.5. Intensity

Although different researchers have provided different conceptual definitions of jump (plyometric) training intensity [80,81,82], including operational (quantitative-objective) attempts [82,83,84], contrasting findings have emerged. Indeed, the intensity of jump training is not easy to define. In line with this, to the authors’ knowledge, the effects of jump training intensity on soccer players’ physical fitness has been spuriously reported in the scientific literature [21]. Moreover, ~34% of the studies conducted in soccer players did not clearly report the intensity of the applied jump drills. When the intensity was reported, in several cases, the description of how intensity was achieved was difficult to interpret or was reported using highly varied criteria, such as jump height or distance, reactive strength index, optimal power, percentage of one-repetition maximum, voluntary effort, velocity, rate of execution, force, rating of perceived exertion, or a mixture of these factors.

Among those studies that reported the exercise intensity, 98% used some form of maximal-intensity prescription including individualized maximal intensity prescription [21,85,86] (e.g., force–velocity profile; reactive strength index). However, no consensus emerged from these studies regarding a clear evidence-based definition (either conceptually or operationally) of jump training intensity for different jump exercises.

Future research should strive to determine optimal jump training intensity markers for soccer players. Such approaches may include laboratory-based techniques (e.g., force platforms) [83,87,88] to assess kinetic and kinematic markers of intensity (e.g., ground reaction force) in different jump exercises. In addition, electromyography [89,90] might offer some value in assessing neuromuscular activation as a potential intensity marker. Furthermore, field-based approaches (e.g., perceived exertion) [21,91,92,93,94] may be particularly well-suited for practitioners to provide insights regarding the role of jump training intensity on soccer players’ physical fitness.

In general, maximal to near-maximal intensity jump training seems safe and, in most instances, is probably necessary to achieve desirable adaptations (e.g., physical fitness) in soccer players. Of course, sound movement competency is also needed, particularly during jump landings since this poses a greater risk of injury in comparison to propulsion/take-off (i.e., kinetic and kinematic landing variables are associated with risk of injury). Several field-based markers of intensity appear to be used within the literature including vertical reactive strength index, jump height, jump distance and rating of perceived exertion, among others.

From a practical (and safety-based) approach, jump training intensity can broadly be defined as maximal for the different types of jump exercises to be performed. However, maximal intensity might involve a reduced contact time from a 60 cm drop landing for an experienced soccer player, or a maximal effort from a 20 cm drop with a strict focus on proper landing mechanics for those with limited jump training background. In this sense, markers of intensity would vary depending on factors such as the participant characteristics (e.g., inexperienced youth players vs. experienced senior players) or training program aims. As individuals accumulate training experience and competency, the tasks may become more complex/demanding with intensity maintained at (near) maximal levels. According to a model previously proposed [95], jump training progression associated with intensity might involve increases in exercise complexity and competence whilst also maintaining maximal effort.

#### 3.3.6. Inter-Repetition Recovery

Most of the included studies (68%) in this review did not provide a clear description of inter-repetition rest interval. Several studies did not incorporate an inter-repetition rest interval as jump drills involved repeated (cyclic) jumps (e.g., rope jump). Among those studies reporting inter-repetition rest intervals, a range from 1 to 60 s was noted.

Most jump-training studies in soccer players reported the use of some form of maximal intensity and after a maximal jump effort, some rest time may be needed to repeat another movement with similar effort. An inter-repetition rest interval of 15 s has been shown to be equally effective compared with longer (i.e., 60 s) intervals to fully recover vertical jumping ability and biomechanical-related parameters (e.g., applied force) [96]. Thus, from a practical perspective, inter-repetition rest intervals of ~15 s may be enough to allow soccer players to recover between jumping efforts. However, several of the included studies in this review reported inter-repetition rest intervals of <15 s whilst observing significant improvements in youth and adult soccer players’ physical fitness. In this sense, relatively shorter inter-repetition rest intervals (i.e., <15 s) may still allow soccer players to attain significant improvements in physical fitness during a jump training program.

Despite the above, no study involving soccer players has addressed the effects of different inter-repetition rest intervals on subsequent physical fitness adaptations. This is a key issue that merits further attention. It may also be of particular relevance when comparing adult and youth soccer players given the ability of younger populations to recover more quickly following maximal or near maximal efforts [97,98].

#### 3.3.7. Inter-Set and Inter-Exercise Recovery

From the included studies, 38% did not report information relating to inter-set and inter-exercise recovery. Among those studies that did report such information, effective recovery intervals varied from 30 to 300 s, and included self-regulated recovery periods. Of note, the required rest period may depend on a myriad of factors such as the duration of the effort, the intensity of the effort and the participant’s specific characteristics [24,98,99]. In this sense, the various type of jump drills and the wide range of participant characteristics (e.g., age; sex, physical fitness level) may explain the wide variety of rest intervals used in the included studies.

However, current available evidence regarding the most effective recovery interval is very limited. Indeed, to the authors’ knowledge, only two studies have analyzed the effect of inter-set and inter-exercise recovery on soccer players’ physical fitness [24,99], both of which involved youth male players. Overall, it seems that both relatively shorter (e.g., ~30 s) and longer (e.g., ~120 s) inter-set rest intervals allow significant physical fitness improvements in youth male soccer players during jump training programs. However, with increasing player age (i.e., greater biological maturity), longer recovery intervals may be preferable in order to maximize improvements in physical fitness (e.g., linear sprint, jumping). Accordingly, sufficiently long rest times would be necessary to maximize jump-training-induced adaptations, especially among those more mature adult participants.

Notably, the effects of passive compared to active recovery strategies were not acknowledged in any of the included studies. Considering the potential of some recovery strategies on athletic performance [100], this would be a relevant area for future research. Similarly, the configuration of recovery time (e.g., cluster vs. traditional set) would also be a relevant area of further research inquiry [101,102,103]. For example, the use of inter-repetition recovery might reduce the need for prolonged inter-set rest intervals, although this has not been previously explored in soccer.

#### 3.3.8. Inter-Session Recovery

From the included studies, 29% did not report information related to the inter-session recovery period. Among those studies that did report such information, effective recovery intervals varied from 24 to 168 h. The most commonly used period was 48 to 72 h (i.e., for those studies that applied 2–3 sessions per week). Even though practical reports recommend that jump training sessions should not be performed on consecutive days (e.g., high-impact plyometrics) [104,105], several interventions demonstrated physical fitness improvements among soccer players using inter-session recovery periods of just 24 h. Moreover, youth soccer players improved physical fitness equally after jump training with either 24 or 48 h of inter-session recovery [23,62]. Similar results were found in amateur female soccer players that completed one or two jump training sessions per week (i.e., 48–120 vs. 168 h of inter-session rest) [18] and in futsal players that performed one or two sessions per week (i.e., 48 vs. 168 h of inter-session rest) [56]. In another study [74], one group of youth male soccer players underwent an inter-session recovery period of 168 h (recovery index [i.e., hours.jump^−1^] = 1.59), while another group underwent 84 h (recovery index = 0.39 h.jump^−1^) with similarly significant improvements noted in athletes’ physical fitness (e.g., jumping; sprinting; change-of-direction speed).

Factors such as training experience, habituation to jump training, age (i.e., maturity status), jumping intensity, type of surface (e.g., sand vs. wood) and recovery strategy (e.g., passive vs. massage) could be considered when rest periods between sessions are assessed [23,97,98,100,106,107]. Overall, it seems that when the total load is rather moderate (i.e., ~100–200 jumps per week, using maximal or near maximal intensity) an inter-session rest of 48 up to 168 h seems effective. In some instances (e.g., low-intensity and/or low-volume jump training sessions), a recovery period of 24 h may be adequate. However, further research efforts are needed to identify optimal inter-session recovery for soccer players, particularly during congested periods of training/competition. In the meantime, practitioners might use some potential field-based markers of recovery in order to assess athletes’ recovery, such as force–velocity profile [108], vertical jump height [107], among others.

#### 3.3.9. Progressive Overload

Progressive overload implicates a gradual increase in the stress placed upon the body during exercise training and is considered to be one of the fundamental principles of training prescription, particularly in relation to resistance training, including plyometric-jump training [109]. Although some of the included studies used a one-dimensional form of overload for soccer players (e.g., volume [35%], technique [2%], and intensity based [6%]), a considerable portion (35%) of the studies combined two or three individual overload techniques during the training program. Of note, ~17% of the included studies did not incorporate an element of progressive overload. Although a non-progressive overload program can be effective in some groups of participants, long-term interventions should ideally adopt a periodized, progressive overload approach. However, the role of rate of volume progression during jump training on soccer players’ adaptations have not been addressed in the research literature thus far. Despite this, when a progressive volume-based overload has been applied [20,73,75] the rate of progression was observed to vary between 10 and 60 jumps per week and was demonstrated to be effective (and safe) to improve soccer player’s physical fitness.

Overall, although progressive overload may not be needed in the short-term (e.g., ≤7 weeks), some form (e.g., volume; intensity; technique; a mixed approach) of progressive overload would likely be needed during longer-term interventions in order to induce ongoing adaptations (e.g., physical fitness) in soccer players. This is in line with the principles of long-term athletic development models [110,111,112], including those in the field of jump training [95]. From a volume perspective, a rate of progression of 10–60 jumps per week or ~10% per week seems effective (and safe) to improve soccer players’ physical fitness. Of course, other forms of progression (e.g., intensity; technique; mixed approaches) warrant further investigation beyond the simple manipulation of volume. From a practical perspective, given the relationship between changes in jump training load and changes in perceived exertion, the assessment of soccer players rating of perceived exertion may offer some value to control the adequate increase in progressive overload [92,113,114].

#### 3.3.10. Taper to Optimize Adaptations after Jump Training

The expected improvements in sports performance from tapering strategies (e.g., programmed reduction in training loads close to competitions [115]) may be related to distinct physiological and metabolic mechanisms such as a more favorable anabolic milieu, improved histological or contractile characteristics of muscles, and increased neuromuscular efficiency [115,116,117]. A recent meta-analysis reported the effects of tapering strategies applied to jump training [26] with 11 studies that included soccer players. From the 11 studies, seven applied a tapering period of ≤7 days whilst the remaining studies applied a period of >7 days. Regarding the magnitude of the taper, eight studies applied a reduction in training volume (>40%) whilst the other three studies applied a reduction ≤40%. The meta-analysis observed similar significant vertical jump improvements after different tapering strategies in terms of volume, taper duration and the total number of jumps completed during the training program (i.e., >1000 total jumps or <1000 total jumps). Although the optimal tapering strategy is yet to be investigated, a meta-analysis (involving athletes from different sports, and different training methods) indicated that a taper phase during which training volume is reduced (provided intensity is maintained) seems to be the most efficient strategy to maximize competitive performance in top-level athletes [118]. Of note, although some studies with soccer players applied tapering strategy during jump training, such studies compared tapering against no tapering on players’ adaptations (e.g., physical fitness). In other words, no jump training study in soccer players has attempted to compare different modes of jump training tapering on adaptations. This is a relevant future line of research in this field. Additionally, determination of the minimal dose to retain adaptations is also a relevant future line of research.

#### 3.3.11. Type of Surface

The type of training surface used for jump training may affect the speed of the stretch-shortening cycle that is performed (e.g., fast vs. slow), alongside other biomechanical and physiological variables [119,120,121], potentially leading toward different adaptations. Indeed, from the included studies in this review, the type of surface used had a significant effect on soccer players’ physical fitness outcomes (e.g., linear sprint; jumping), including male youth [27,63,122,123,124], adult players [125].

Although the optimal type of surface is yet to be determined, considering the potential relevance of the type of surface used during jump training on soccer players’ adaptations (e.g., physical fitness and stiffness), it was somewhat surprising that the surface type was not clearly reported in ~60% of the included studies. Among those studies reporting the surface type, grass-type surface was most common (~22%), likely associated to the specificity of the grass surface where soccer players usually train and compete. The remaining studies reported tartan-turf-synthetic surface (n = 6), unstable surface (n = 5), water (n = 2), sand (n = 1), gym-mats (n = 2), a combination of different surfaces (n = 5), or a special developed jumping apparatus (n = 3). Noticeably, the quantification of the hardness of the training surface (e.g., by means of the restitution coefficient [velocity after collision/velocity before collision] [126]) was rarely reported.

Considering the potential influence of the aforementioned factors on ground reaction forces and stiffness, among other biomechanical factors and physiological responses [119,120], future studies may devote further attention to these potentially relevant aspects of jump training prescription among soccer players. For practitioners, although a grass surface may be the most sport-specific type of surface for soccer players, depending on the player’s needs, some additional surface types may be used to underpin recover or performance (e.g., softer surfaces to reduce delayed-onset of muscle soreness).

#### 3.3.12. Jump Training Combined with Other Training Methods

Another factor that may be of relevance during jump training is its sequencing with other training methods. Although soccer alone may be effective to improve athletes’ physical fitness [127], sophisticated training-methods may be needed with increased levels of competition. Different training methods have been reported in the scientific literature aimed to improve soccer players’ physical fitness (e.g., linear sprint; jumping; endurance), such as small-sided games [128], high-intensity interval training [129], endurance training [6], sled training [130], neuromuscular training [131], core training [132], isoinertial training [133], balance training [134], soccer-specific training (e.g., FIFA 11) [135], altitude-preparation training [136,137], among others [138,139,140]. Commonly, one or more of the aforementioned training methods should be sequenced with jump training.

One study with youth male soccer players [22] noted that when jump training was incorporated at the beginning of regular training sessions (i.e., immediately after the warm-up), greater physical fitness improvements were noted compared to when the same jump training was applied at the end of the regular training sessions. Another study reported that jumping performance is impaired after soccer-specific activity and fast stretch-shortening cycle tasks suffer the greatest [141]. Although currently there is no evidence regarding the optimal combination of jump training with other training methods for soccer players, the information derived from the aforementioned studies [22,141] suggests that training methods such as jump training, involving maximal or near maximal short-duration efforts may be applied with greater effect prior to other training methods, such as technical skill training. This is in line with previous recommendations [34], so that jump training exercises are not performed in a fatigued state, particularly those involving high eccentric forces and short ground contact times (e.g., plyometrics) [105].

Although jump training may be regarded as an effective training method for soccer players, rather than an independent entity, the method should be a component of an integrated approach to soccer players’ physical development, which concurrently targets multiple physical fitness qualities and aligns with the goals of long-term physical development strategies.

**Figure 2:**
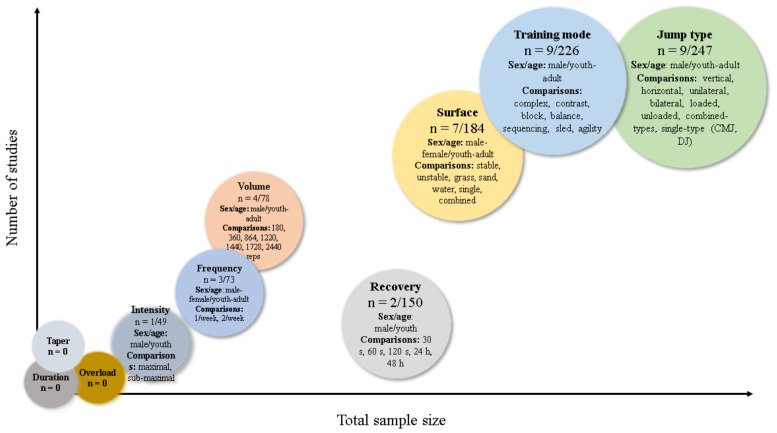
Overview of available experimental literature analysing relevant jump training programming factors in soccer players. Note: bubble size are relative to total sample size (n = number of studies/total sample size).

Figure 2 contains a graphical summary of the identified experimental literature on programming parameters and further methodological characteristics of plyometric-jump training studies in soccer.

### 3.4. Potential Limitations

This review article only included studies written in English. We are aware that this is not in line with international recommendations [142,143,144]. However, previous findings suggested that 99.6% of the plyometric-jump training literature is available in English language [11]. Therefore, if studies in other languages are available, they would constitute a minor portion of the overall number of studies. Nonetheless, future review articles should also focus on articles written in different languages.

Additionally, only one author participated in the selection and coding of the articles, potentially reducing results reliability [145]. However, the selection and coding of the articles was performed by an experienced reviewer, a methodological approach that, compared to those performed by less experienced reviewers, could still be appropriate [145]. Indeed, the reviewer in charge of the selection and coding of the articles might be considered as the most experienced reviewer in the world in this particular topic (i.e., plyometric exercise, https://www.expertscape.com/ex/plyometric+exercise; accessed on 1 May 2022). Nonetheless, a two-step selection process (e.g., conventional double screening; one reviewer and one verifier) is generally recommended [142,143,144].

Of note, our living search strategy in the electronic databases allows to provide updates periodically. As previously recommended [51], such updates are planned every five years, and will not be restricted to language and will be planned to include at least two reviewers in methodological steps such as article selection and coding.

### 3.5. Perspectives for Future Research

Jump training is a popular and convenient method to improve physical preparedness in soccer players. Recently, there has been a proliferation of published articles on the effects of jump training in soccer players. However, there have been relatively few studies conducted in female soccer and master athletes. From the 90 included studies in our review, only twelve involved female players. Furthermore, among those studies conducted in females, only three involved youth females. This contrasts starkly with the relatively greater number of studies conducted in youth males (i.e., 52 from a total of 77). Soccer is a very popular sport around the world, with nearly 270 million people actively playing and a 50% increase in the number of female players observed between 2000 to 2006 [1]. Indeed, a stated aim for the sport is to reach 60 million female players by 2026 [2]. In light of this, research on female players is required to enhance knowledge with regard to a scientific approach to soccer performance in this fast-growing population.

In addition, there is little information available on how jump training programming variables (e.g., intensity; duration; overloading) modulate the effects of jump training programs. Among the 90 included studies, only 35 incorporated an experimental design intended to determine the effectiveness of a given jump training programming factor. Moreover, a key training programming variable such as jump training intensity has been addressed only by one experimental study. Jump training programming variables such as jump type have received more experimental research attention with nine experimental studies involving 247 soccer players. However, given the high number of potential jump training exercises that can be incorporated in a jump program, the effect of most of these exercises has not been properly investigated. Further areas of inquiry may involve the effectiveness of jump training in different periods of the soccer season (e.g., pre-season; off-season; in-season), as this is a common practice [10], with potential impact on physical fitness and injury reduction risk [146]. Nonetheless, empirical evidence is lacking. Additionally, the effects of jump training may vary according to players’ position on the field (e.g., defenders; forwards) and this factor has received little attention in the literature to date. In the future, researchers are advised to conduct jump training studies of high methodological quality (e.g., randomized controlled trials), with more research needed on specific dose–response relationships (particularly in chronic or long-term exercise studies), including duration, frequency, type of jump exercise, volume, intensity, recovery, progressive overload, taper strategies, type of surface, and the effects of jump training combined with other training methods.

## 4. Conclusions

In conclusion, based on the limited available research, it seems that a dose of 7 weeks, with 1–2 sessions per week, with ~80 jumps (specific of combined types) per session or 140–240 per week, using near-maximal or maximal effort intensity and proper technique, with inter-repetition rest (if needed) <15 s, inter-set rest of ≥30 s, inter-session recovery of ≥24–48 h, using progressive overload (e.g., 10% weekly increase in number of jumps) coupled with taper strategies (e.g., reduced volume and intensity maintained) when required, using appropriate surfaces such as grass, and applied in a well-rested state (i.e., non-fatigued), when combined with other training methods, would increase outcome of effective and safe plyometric-jump training interventions aimed at improving soccer players physical fitness. This review article described the effectiveness of plyometric-jump training by taking the impact of specific programming parameters into account in different soccer populations (youth, adult, male, and female). Yet, more original research is needed to elucidate optimal programming parameters for plyometric-jump training in soccer according to fitness level.

## Figures and Tables

**Table 1:** Search strategy (search syntax) for each database.

**Date of the search**	April 2017	May 2019	September 2021
**Databases**	PubMed	PubMed, WOS (Core Collection), Scopus	PubMed, WOS (Core Collection ^a^), Scopus
**Keywords**	“plyometric”, “training”	“ballistic”, “complex”, “cycle”, “explosive”, “force”, “plyometric”, “shortening”, “stretch”, “training”, “velocity”	“ballistic”, “complex”, “cycle”, “explosive”, “force”, “jump”, “plyometric”, “power”, “shortening”, “stretch”, “training”, “velocity”
**Applied database fields used during the search**	All	PubMed: all WOS: all Scopus: title, abstract, keywords	PubMed: all ^b^ WOS: all ^b^ Scopus: title, abstract, keywords ^b^
**Restrictions for the search**	None	None	None
**Examples of the search strategy (syntax)**	Pubmed: “plyometric exercise” [MeSH Terms] OR (“plyometric” [All Fields] AND “exercise” [All Fields]) or “plyometric exercise” [All Fields] OR (“plyometric” [All Fields] AND “training” [All Fields]) OR “plyometric training” [All Fields] WOS: (All = (plyometric)) and All = (training) Scopus: Title-Abs-Key (plyometric AND training)		

^a^: except for the keywords “jump” and “power” searched in all WOS databases. ^b^: except for the keywords “jump” and “power” searched in the database field TITLE.

**Table 2:** Summary of the characteristics of soccer players in jump training studies.

	Adult Male	Youth Male	Male Total	Adult Female	Youth Female	Female Total
No. of studies	25	52	77	8	3	11
Sample size (median) per study group	10	13		10	11	
Age (yrs)	18–25 ^§^	9.5–17.8		18.3–24.3	13.4–16.5	
Body mass (kg)	60.7–83.1	31.0–74.5		54.9–61.1	50.8–61.5	
Height (cm)	161–184	130–180		158–167	162–167	
No. of studies that included a randomization procedure	22	51	73	6	1	7
No. of studies with control groups	14	34	48	6	1	7
No. of studies describing training interventions *	18	29	47	6	0	6
No. of studies where the fitness of participants was:						
High	10	6	16	1	1	2
Moderate	12	45	57	6	1	7
Recreational	3	1	4	1	1	2
No. of studies where players had experience with jump training	7	29		4	2	
No. of studies with in-season interventions	15	39		6	2	

*: described the duration, frequency, intensity, type of exercises, sets, and repetitions of the jump training program. ^§^: minimum-maximum range values.

## Data Availability

The datasets generated during and/or analyzed during the current study are available from the corresponding author on reasonable request.
